# Overexpression of PD2 leads to increased tumorigenicity and metastasis in pancreatic ductal adenocarcinoma

**DOI:** 10.18632/oncotarget.6580

**Published:** 2015-12-12

**Authors:** Arokia Priyanka Vaz, Shonali Deb, Satyanarayana Rachagani, Parama Dey, Sakthivel Muniyan, Imayavaramban Lakshmanan, Saswati Karmakar, Lynette Smith, Sonny Johansson, Subodh Lele, Michel Ouellette, Moorthy P. Ponnusamy, Surinder K. Batra

**Affiliations:** ^1^ Department of Biochemistry and Molecular Biology, University of Nebraska Medical Center, Omaha, NE, USA; ^2^ Department of Biostatistics, University of Nebraska Medical Center, Omaha, NE, USA; ^3^ Department of Pathology and Microbiology, University of Nebraska Medical Center, Omaha, NE, USA; ^4^ Department of Internal Medicine, Division of Gastroenterology and Hepatology, University of Nebraska Medical Center, Omaha, NE, USA; ^5^ Eppley Institute for Research in Cancer and Allied Disease, University of Nebraska Medical Center, Omaha, NE, USA

**Keywords:** PD2, CSC, c-Myc, PDAC

## Abstract

Pancreatic differentiation 2 (PD2), an important subunit of the human PAF complex, was identified after differential screening analysis of 19q13 amplicon, and its overexpression induces oncogenic transformation of NIH3T3 cells, hence raising the possibility of a role for PD2 in tumorigenesis and metastasis. To test this hypothesis, we analyzed here the functional role and clinical significance of PD2 in pancreatic ductal adenocarcinoma (PDAC) and its pathogenesis. Using immunohistochemical analysis, we found that PD2 is detected in the acini but not in the ducts in the normal pancreas. In human PDAC specimens, PD2 was instead primarily detected in the ducts (12/48 patients 25%; *p*-value < 0.0001), thereby showing that PDAC correlates with increased ductal expression of PD2. Consistently, PD2 expression was increased in telomerase-immortalized human pancreatic ductal cells (HPNE cells) modified to express the HPV16 E6 and E7 proteins, whose respective functions are to block p53 and RB. In addition, ectopic expression of PD2 in PDAC cells (Capan-1 and SW1990) led to increased clonogenicity and migration *in vitro*, and tumor growth and metastasis *in vivo*. Interestingly, PD2 overexpression also resulted in enrichment of cancer stem cells (CSCs) and upregulation of oncogenes such as c-Myc and cell cycle progression marker, cyclin D1. Taken together, our results support that PD2 is overexpressed in the ducts of PDAC tissues, and results in tumorigenesis and metastasis via upregulation of oncogenes such as c-Myc and cyclin hence D1 implicating PD2 upregulation in pancreatic oncogenesis with targeted therapeutic potential.

## INTRODUCTION

Pancreatic ductal adenocarcinoma (PDAC) is the fourth leading cause of cancer-related deaths in USA and projected to be the second leading cause of cancer deaths by 2020 [1]. According to the American cancer society, PDAC incidence to mortality ratio is nearly one [2]. The poor survival rate of PDAC patients is due to late diagnosis, early metastasis [3] and resistance to current therapies. Nearly 70 percent of PDAC deaths are due to metastasis to distant organs and hence recent research has been focused towards the identification of molecular targets to prevent metastasis and disease recurrence. Emerging reports have shown that cancer stem cells (CSCs) contribute to the epithelial to mesenchymal transition (EMT) phenotype, metastasis [4] and disease recurrence. The high mortality rate of PDAC patients makes it imperative to study the key oncogenic molecules essential for the maintenance of CSC population that causes PDAC progression and metastasis.

Early genetic alterations and inactivation of various gatekeeper genes involved in cell cycle regulation such as p16^INK4A^/p14^ARF^ and p53 are reported to cause PDAC [5]. Likewise, epigenetic alterations such as histone modifications and DNA methylation are also shown to contribute to PDAC development [6]. Moreover, recently proven CSC hypothesis in various cancers including PDAC ascertains that there are specific alterations, including overexpression of CD133, Sox2, CXCR4 and Pancreatic differentiation 2 (PD2) in a minor subpopulation of cancer cells, which are responsible for chemotherapeutic resistance and tumor recurrence [7–9]. Further, it has been shown that acinar to ductal metaplasia (ADM) plays a major role in PDAC pathogenesis [10, 11]. Cumulatively, many such alterations could give rise to PDAC. One such molecule which plays a role in cell cycle regulation, mouse embryonic stem cell (mESCs) maintenance, oncogenesis, histone methylation, chromatin remodeling, ADM, CSC maintenance and drug resistance is PD2 [9, 12–16]. PD2 is the human homologue of yeast Paf1 and is the core component of the human PAF complex, which consists of other subunits, such as hCtr9, hCdc73, hLeo1 and hSki8 [17]. PD2 was identified as a potential oncogene and its overexpression led to the oncogenic transformation of NIH3T3 fibroblast cells [14]. In addition, PD2 was found to assist in the maintenance of mESCs via interaction with Oct3/4 [13]. PD2 has also been shown to be involved in histone methylation and chromatin remodeling in PDAC cells [12, 15]. Further, our recent findings demonstrated the role of PD2 in the maintenance of pancreatic CSCs and drug resistance [9].

Here, we have investigated the functional role and clinical significance of PD2 in PDAC and its pathogenesis. We have demonstrated the expression of PD2 in tissues obtained from PDAC patients and normal pancreas. Expression of PD2 was observed in various PDAC cell lines and in telomerase-immortalized human pancreatic ductal cells. By using PD2 overexpressed Capan-1 and SW1990 PDAC cells, we have shown that PD2 increases PDAC cell growth and tumorigenesis both *in vitro* and *in vivo*. In addition, we have shown that overexpression of PD2 leads to an increase in side population (SP)/CSCs and an enhancement in signaling by downstream molecules and its implications in metastasis. Altogether, our study showed for the first time that PD2 promotes tumorigenesis in pancreas, as presented below.

## RESULTS

### Expression of PD2 is high in human PDAC and metastatic tissues

**Figure 1:**
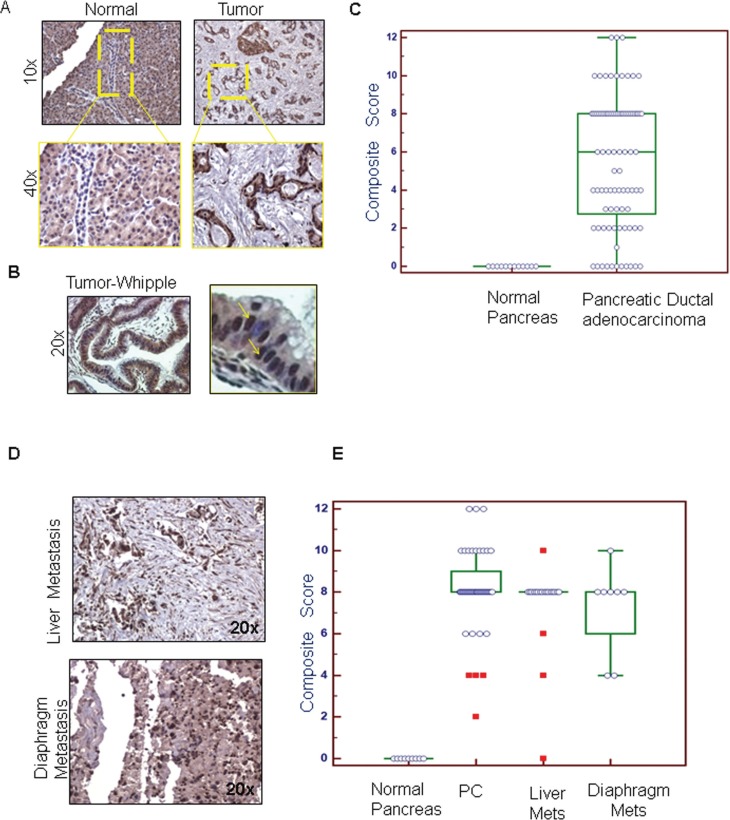
Immunohistochemical comparison of the expression of PD2 in normal pancreas, PDAC and metastatic tissues (**A**) The expression of PD2 was investigated in the normal and the PDAC tissues. PD2 expression was absent in the ducts of the normal pancreas whereas positive staining was observed in the pancreatic ductal adenocarcinoma (yellow box). There is a significant increase in the expression of PD2 in the PDAC tissues when compared to the normal pancreas. (**B**) The expression of PD2 was elevated in tissue samples obtained from patients who underwent Whipple procedure. The magnified image in the right panel indicates overexpression of PD2 in few cells. (**C**) The staining score and the intensity scores were multiplied to obtain the composite score which was subsequently represented as box plots. The box plot denotes the PD2 expression in the ductal region of PDAC tissue in X-axis and its composite score in Y-axis. (**D**) PD2 expression in the metastatic tissues was examined. (**E**) The staining score and the intensity score were multiplied to obtain the composite score which was represented in the box plots as shown in the bottom panel. The box plot denotes the total no. of spots in the primary and the metastatic tissues along with the composite scores on the *y* axis. Both the primary tissues (1A) as well as the metastatic tissues had significantly higher CS than the normal tissues.

To investigate the clinical significance of PD2 in PDAC pathogenesis, PD2 expression pattern was analyzed in normal and PDAC tissues. Immunohistochemistry (IHC) analyses revealed that the expression of PD2 was significantly higher in PDAC ducts when compared to the normal duct (Figure 1A, 1B). A negative control was included to confirm that the antibody had specific staining (data not shown). The PD2 immunostaining was analyzed in a total of 89 spots which were comprised of 48 PDAC, 32 metastasis to different organs (20 liver spots, 8 diaphragm spots, and 4 small bowel spots), and 9 normal tissue sections. Approximately 8.33% of ducts in Pancreatic Ductal Adenocarcinomas (PDACs) showed weak PD2 staining (composite score (CS) 1–4), 66.67% moderate staining (CS 5–8), and 25% strong staining (CS 9–12). The normal pancreatic tissues showed no specific staining in the ducts, whereas the acini stained moderately positive. The median CS (CS = 8) was higher in PDAC tissues when compared to normal pancreas as seen by Wilcoxon rank sum test with a *p*-value < 0.0001 (Figure 1C). Similarly, all the metastatic sites such as liver and diaphragm showed higher expression (CS = 8) of PD2 (Figure 1D) when compared to normal tissues (CS = 0). PDAC tissues obtained from the patients who underwent Whipple procedure revealed that 33 tissues (70%) stained positive for PD2, whereas, 10 tissues scored negative (*p* = 0.037). The CS of PDAC tissues obtained from RAP and Whipple samples are combined and plotted together along with the CS of metastatic sites present in the tissue arrays (Figure 1E).

### PD2 is differentially expressed in PDAC cells

**Figure 2:**
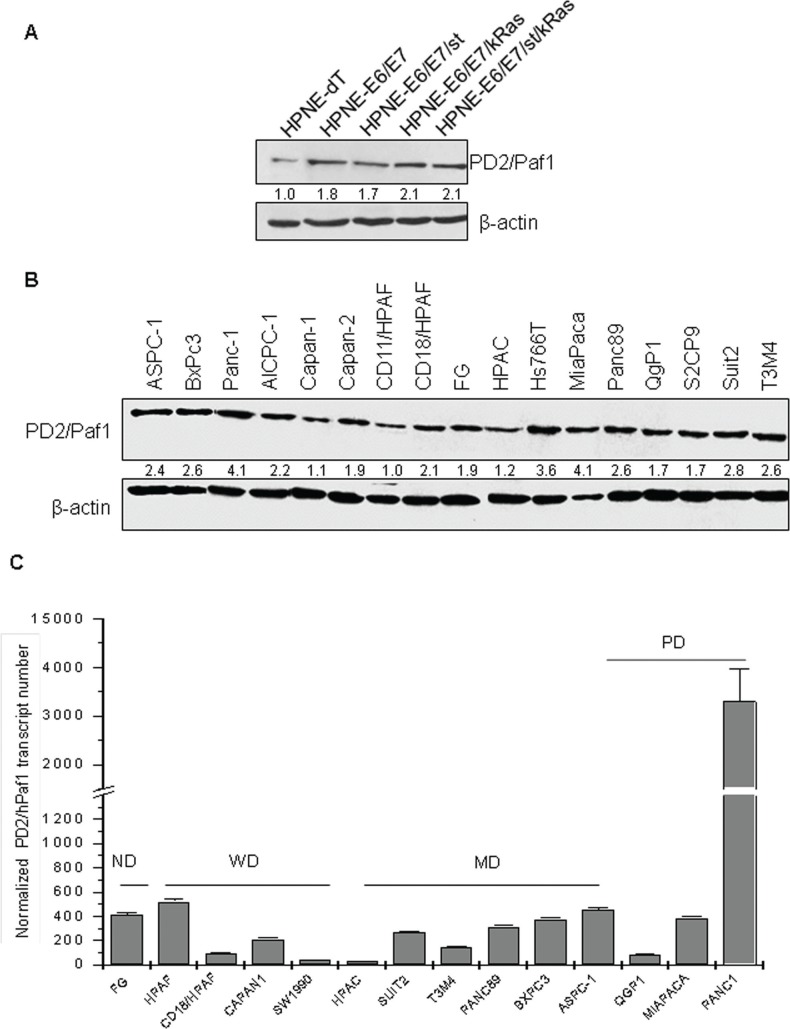
PD2 expression level in a panel of PDAC cell lines using western blot and real time PCR (**A**) Protein lysates from the HPNE progression model were resolved on a 10% SDS-PAGE gel. PD2 expression was observed in all the cell lines. The expression of PD2 was higher in the oncogenes transformed cell lines when compared to the parental HPNE. (**B**) Protein lysates from 17 PDAC cell lines were resolved on a 10% SDS-PAGE gel. The expression of PD2 was observed in all the cell lines when incubated with anti-PD2 antibody (upper panel). For loading control, membranes were stripped and re-probed with anti-β-actin (lower panel). PD2/hPaf1 presents a band of 80 kDa. (**C**) hPaf1 expression was analyzed by real time-PCR on 14 PDAC cell lines, SW1990, AsPC-1, Panc-89, T3M4, CD18/HPAF, Panc-1, MiaPaCa, Suit2, FG-Colo, Qgp1, HPAC, BxPC3, Capan-1, Capan-2. GAPDH expression was used as an internal reference. There is a 30-fold overexpression of PD2/hPaf1 in the poorly differentiated Panc-1 cell line, correlating with its gene amplification. MiaPaCa, another poorly differentiated cell line, and ASPC-1, a moderately to poorly differentiated cell line, showed moderate to high PD2/hPaf1 expression, thus indicating that PD2/hPaf1 is expressed at higher levels in poorly differentiated cells as compared to the well differentiated cells.

The expression of PD2 was analyzed in a line of telomerase-immortalized human pancreatic ductal cells (hTERT-HPNE) and its transformed variants produced by stepwise introduction of oncogenes, including HPV16 E6 and E7 proteins (E6/E7), SV40 small t antigen (st), and oncogenic K-Ras (Kras) [18]. Interestingly, immunoblot analyses revealed that the expression of PD2 was higher in the HPNE cells expressing the E6 and E7 proteins, whose respective functions are to block the tumor suppressors p53 and RB (Figure 2A). In addition, immunoblot analysis (Figure 2B) and real time RT-PCR (Figure 2C) revealed that PD2 was ubiquitously expressed in all PDAC cell lines. PD2 expression level was higher in poorly-differentiated PDAC cells (Panc-1, MiaPaCa and Hs766T), and moderate to weak in both well-differentiated (Suit2, CD18/HPAF, CD11/HPAF, Capan-1, CD11/HPAF, Capan-2, S2CP9) and moderately-differentiated (Panc-89, BxPC3, T3M4, AsPC-1) (Figure 2B) PDAC cell lines.

Quantitative analysis by qRT-PCR revealed that the expression of PD2 transcript was significantly higher in the poorly-differentiated cell line, Panc-1 with a 30-fold overexpression as compared to the other cell lines (Figure 2C). These data retrospect to the discovery of PD2 by a differential screening analysis which revealed that PD2 was 30 fold overexpressed in poorly differentiated cell line, Panc1 when compared to well differentiated cell line CD11 [14]. Another poorly-differentiated cell line MiaPaca also showed a high level of PD2 expression at RNA level, which correlated with the protein expression (Figure 2C vs. 2B). These results reveal a differential expression pattern of PD2 in PDAC cell lines, thereby suggesting a potential association between PD2 expression and the differentiation of PDAC cells. GAPDH was used to normalize the transcript levels.

### PD2/hPaf1 gene contains no mutations

PDAC is characterized by inherited and acquired mutations in a variety of genes [19]. PD2 is part of the 19q13 locus, containing the AKT2 gene, which is amplified in 10% of PDACs [20]. We thus wanted to investigate mutation in the PD2 gene, apart from the gene amplification that occurs in PDAC. The PD2 coding region was amplified in 2 overlapping fragments from 13 different PDAC cell lines (Panc-1, MiaPaCa, BxPC-3, Capan-1, HPAF, SW1990, AsPC-1 T3M4, Capan-2, CD18, CFPAC-, Panc-89, Colo357) (Supplementary Figure S1). The amplified regions were then sequenced and analyzed by Mutation Surveyor^™^ version 3.01 (SoftGenetics, LLC). Our results revealed that there were no mutations in the coding region of PD2 in any of the PDAC cell lines (Supplementary Figure S1).

### Overexpression of PD2 increases PDAC cell growth

**Figure 3:**
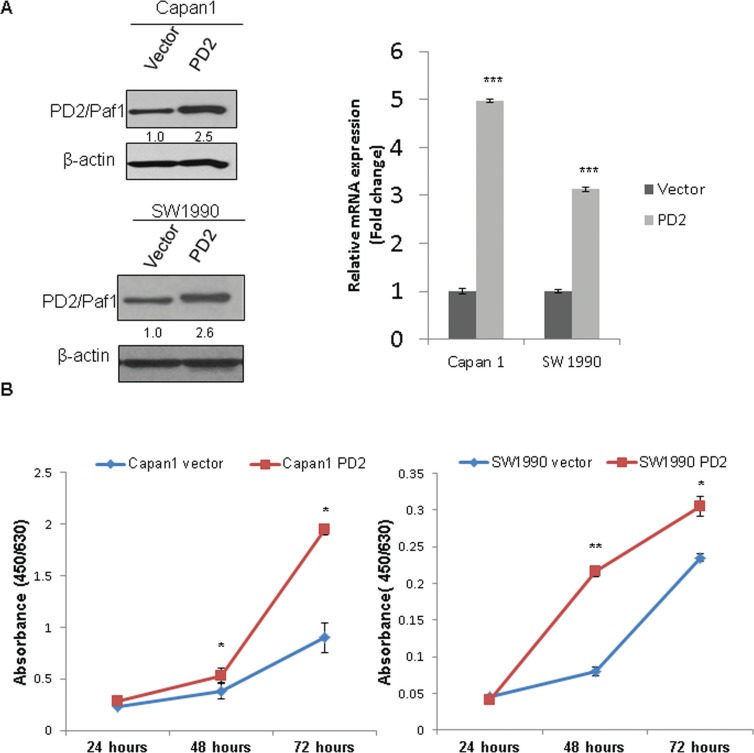
Ectopic overexpression of PD2 led to increased PDAC cell growth (**A**) The expression of PD2 was analyzed in the Capan-1 and SW1990 transfected cells using western blot. A 2.5 fold and > 3 fold overexpression of PD2 was observed at protein and RNA levels respectively in Capan-1 and SW1990 when compared to the vector transfected cells. β-actin served as a loading control (*p*-value < 0.0005). (**B**) PD2 overexpression led to increased growth in cancer cells. Growth kinetics was performed in PD2 overexpressing and control Capan-1 and SW1990 cells. The cell growth rate was recorded on days 1, 2, and 3. PD2 overexpression led to increased growth of cancer cells in the overexpressed Capan-1 and SW1990 cells when compared to the control cells (*p*-value < 0.05).

To investigate its tumorigenic role, PD2 was ectopically overexpressed in Capan-1 and SW1990 PDAC cells. FACS sorted GFP positive cells were validated by both immunoblot and qRT-PCR for PD2 expression. We could find at least, 2.5 fold overexpression by immunobloting and > 3 fold overexpression at RNA level in both Capan-1 and SW1990 PDAC cells as compared to the vector control cells (Figure 3A). As shown in Figure 3B, PD2 overexpression led to the increased growth of PDAC cells as compared to the vector transfected control cells, with a *p*-value of < 0.01 (Figure 3B).

### PD2 overexpression increases clonogenic and migratory abilities of PDAC cells

**Figure 4:**
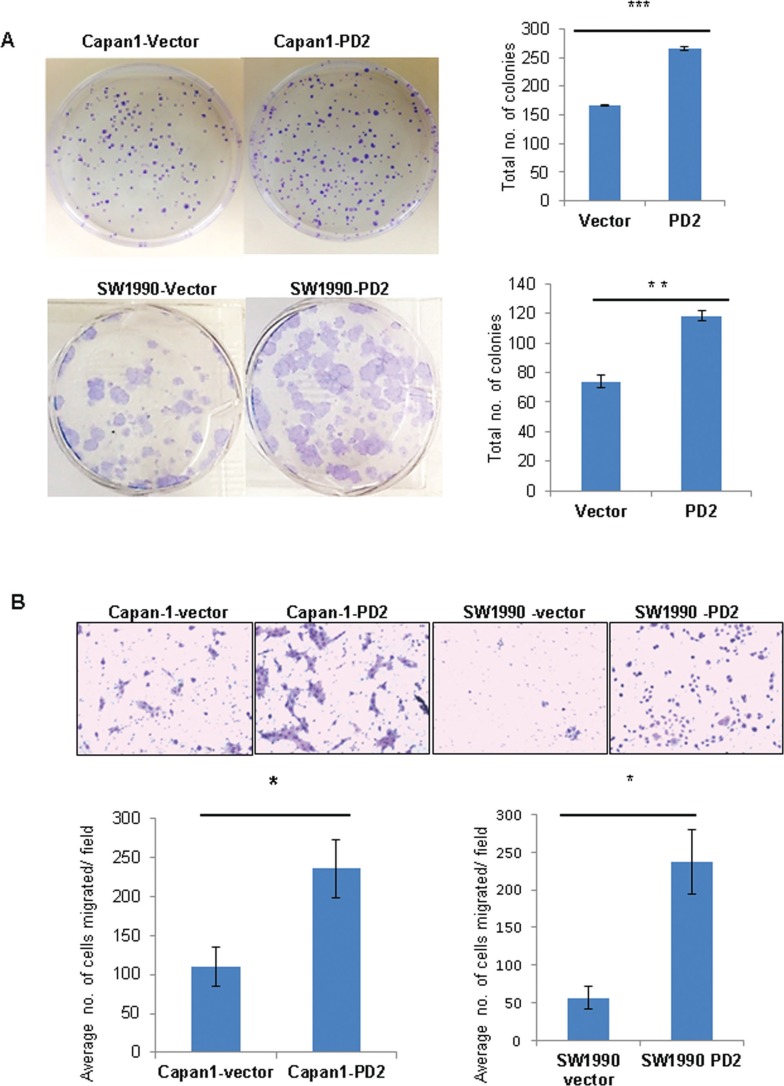
PD2 enhances colony forming and migratory potential (**A**) Under anchorage dependent conditions Capan-1-PD2 and SW1990-PD2 cells showed increased colony formation ability when compared to the vector transfected cells. The colonies were stained using crystal violet and photographed. The total number of colonies were counted and averaged. (**B**) PD2 overexpression promotes migration of Capan-1 and SW1990 cells. Overexpression of PD2 led to increased migration potential in Capan-1-PD2 and SW1990-PD2 transfected cells when compared to the vector transfected cells. Cell migration was assessed using trans-well migration chambers. Representative photographs were shown. The total number of migrated cells were counted and averaged. These results are an average of 20 random microscopic fields taken from 2 independent experiments (*p*-value < 0.05 = *p-value < 0.005 = **p-value < 0.0005 = ***).

The *in vitro* tumorigenic potential of PDAC cells upon variation of PD2 expression was analyzed by a clonogenic assay. Ectopic overexpression of PD2 in Capan-1 and SW1990 leads to formation of significantly greater number of colonies compared to the control cells (Figure 4A), indicating that PD2 overexpression increases clonogenic potential. Further, the migratory properties of PD2 overexpressing cells were analyzed both in Capan-1 and SW1990 PDAC cells. Our results show that ectopic overexpression of PD2 led to a significant two-fold increase in migration of PDAC cells (Capan-1 and SW1990) (Figure 4B). These *in vitro* results suggest that PD2 overexpression can lead to increased metastatic potential of PDAC cells.

### PD2 overexpression increases tumorigenicity and metastasis *in vivo*

**Figure 5:**
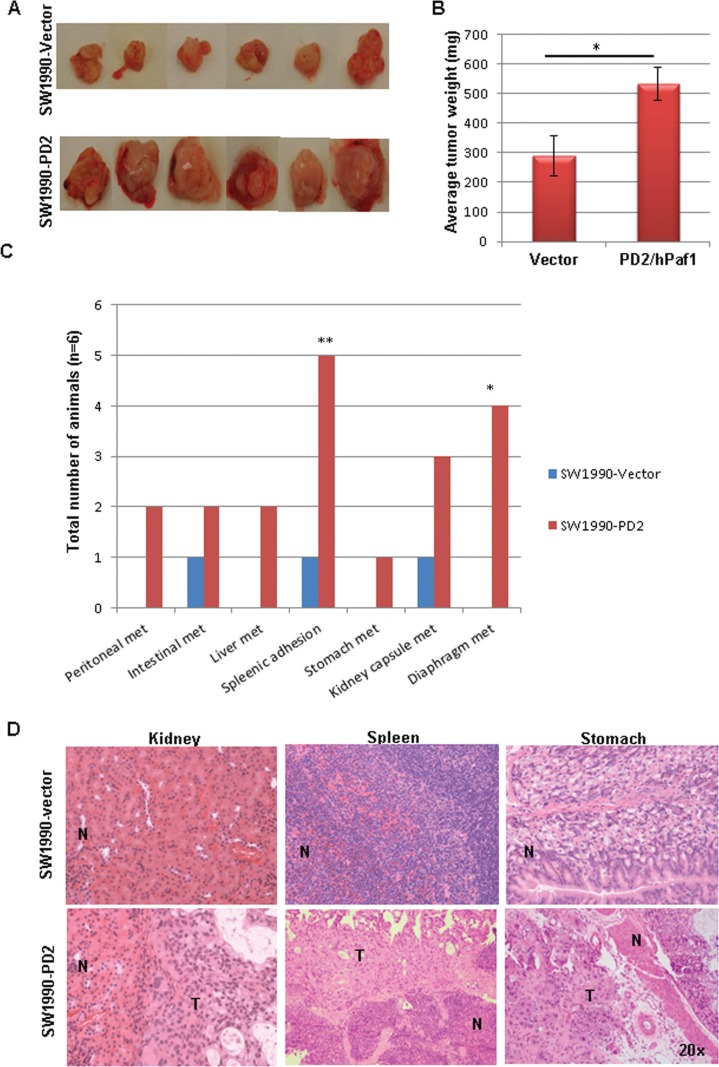
PD2 overexpression promotes tumor growth and metastasis *in vivo* (**A**) Orthotopic implantation of PD2 overexpressed SW1990- PDAC cells show increased tumor forming ability when compared to the vector transfected cells. Photographs represent the tumors which were harvested from both the groups. (**B**) The tumors were resected from mice (*n* = 6) weighed and averaged; the histogram displays the tumor weights of mice that received PD2-transfected cells compared to the mice which received control vector transfected PDAC cells (*p*-value = 0.021). (**C**) Nude mice developed widespread macro-metastases when SW1990-PD2 overexpressing cells were orthotopically injected into the pancreas. There were a significant number of metastatic nodules observed in the spleen (83.3%) and diaphragm (66.7%). *P*-values of less than 0.05 were considered statistically significant. (**D**) Micrographs of H & E staining represent the occurrence of metastasis in kidney, spleen and stomach in the SW1990-PD2 transfected animals.

To determine the effect of PD2 overexpression in tumorigenicity and metastasis *in vivo*, PD2 overexpressing and control cells were orthotopically implanted in the head of the pancreas of nude mice (*n* = 6 per group). Animals were monitored daily and sacrificed 7 weeks after implantation. Primary tumors in the pancreas were resected and gross metastatic lesions were carefully observed in distant organs. PDAC cells with ectopic overexpression of PD2 formed significantly bigger tumors (average weight = 540 mg) when compared to the vector injected PDAC cells (average weight = 290 mg) (Figure 5A, 5B). Interestingly, PD2 overexpression in PDAC cells resulted in metastasis to distant sites such as liver, stomach, kidney capsule, diaphragm, peritoneal wall and intestine. In addition, splenic adhesions were observed in 5/6 animals injected with PDAC cells overexpressing PD2 (Figure 5C). Hematoxylin and eosin staining of the sections from distant organs of nude mice implanted with PDAC cells further verify the metastatic potential of PD2 overexpressed cells (Figure 5D). Similar orthotopic experiments was performed with Capan-1 in a smaller number of animals (*n* = 3 per group) which further strengthen our results that PD2 overexpression resulted in increased tumor formation and metastasis (Supplementary Figure S2).

### Increased SP/CSC population in PD2 overexpressed cells

**Figure 6:**
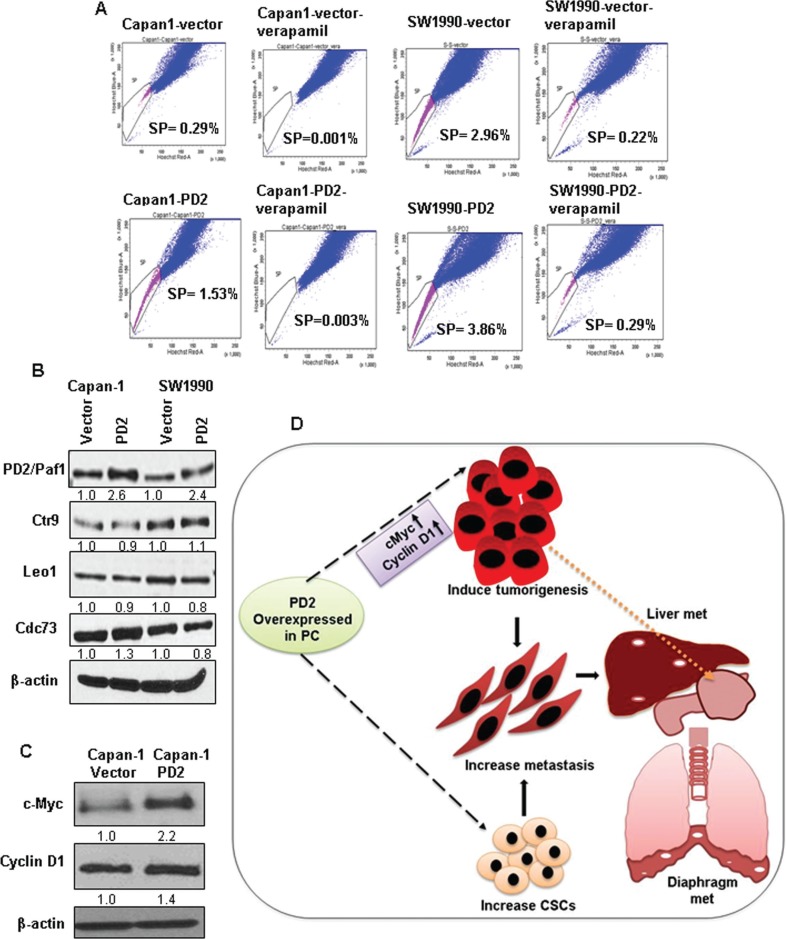
Effect of PD2 overexpression on the enrichment of SP/CSC population and on other PAF1 complex member proteins (**A**) FACS analysis shows a clear increase in the number of SP/CSC population in PD2 overexpressed cells when compared to control cells. A total of 0.29% and 2.96% SP population were observed in Capan-1 and SW1990 vector transfected cell line and 1.5% and 3.86% SP were observed in the PD2 transfected Capan-1 and SW1990 PDAC cells respectively. Verapamil treated cells served as a control for isolating SP cells and it shows a clear decrease in the SP cells (Capan-1-Vector-0.001%, Capan-1-PD2-0.003%, SW1990-Vector-0.216%, SW1990-PD2-0.287%). (**B**) The effect of PD2 overexpression was assessed on other PAF1 complex proteins. PD2 overexpression led to a moderate upregulation of Cdc73 in Capan-1-PD2 cells when compared to the control cells. However, there was no change in the expression of Ctr9 and Leo1. No difference was observed in the expression of PAF complex members in SW1990 cells. (**C**) The expression of c-Myc was found to be two fold upregulated in Capan-1-PD2 cells. However, SW1990 cells lacked the expression of c-Myc. Cyclin D1 was found to be upregulated in Capan-1. (**D**) Schematic representation of the effect of PD2 overexpression in PDAC on tumorigenesis and metastasis. PD2 overexpression results in increased growth of PDAC cells due to upregulation of c-Myc and cyclin D1 which led to increased tumor growth in the pancreas of the nude mice (as indicated by the spotted arrow). Overexpression of PD2 led to metastasis (indicated as the white spotted lesions) to distant organs including liver and diaphragm. Most importantly, the elevated expression of PD2 led to an increase in the CSC population when compared to the control cells.

Our previous study demonstrated that overexpression of PD2 led to the maintenance of SP/CSCs in PDAC [9]. Based on this observation, we performed a retrospective study to examine the effect of PD2 overexpression on the SP/CSC population. Interestingly, we observed that PD2 overexpression led to an increase in SP/CSCs of 5.3 fold in Capan-1 (1.53% vs. 0.29%) and 1.3 fold in SW1990 (3.86% vs. 2.96%) PDAC cells compared to the vector transfected cells (Figure 6A). These results show that PD2 overexpression is associated with the CSC phenotype, thereby corroborating our previous results [9].

### Effect of PD2 overexpression on other PAF complex members

PD2 is a crucial member of the hPAF complex which consists of other members, including hCtr9, hSki8, hLeo1, and hCdc73. Earlier studies revealed that downregulation of PD2 in Panc1 and MiaPaca-2 PDAC cells resulted in the down regulation of PAF complex members hCtr9, hLeo1, hCdc73, but not hSki8 [15]. In Capan-1 cells, we observed that overexpression of PD2 led to the upregulation of hCdc73 alone by 1.3 fold in Capan-1 cells, with no observed difference in the expression level of other subunits (Figure 6B). In SW1990 cells, no significant difference was observed in the expression of the PAF complex subunits (Figure 6B).

### PD2 increases the expression of oncogenes and affects downstream signaling

To investigate the perturbations in oncogenic signaling caused by PD2 overexpression, we examined the expression of c-Myc, a critical regulator of the G1/S transitions involved in tumorigenesis in PDAC [21]. Interestingly, we found that PD2 overexpression leads to the up-regulation of c-Myc in Capan-1 cells. PD2 overexpression also led to increased cyclin D1 expression, a downstream target of c-Myc and central regulator of the G1/S transition (Figure 6C). These results suggest that PD2 overexpression is causing an elevated expression of c-Myc and cyclin D1 either by localizing at their promoters or by activating other transcription factors upstream of these genes.

### Microarray analysis reveals differential gene expression pattern

In order to see the differential expression of genes in the PD2 overexpressed cells, microarray was performed using vector control and PD2 overexpressing Capan-1 cells. The analysis of the microarray data revealed that genes such as BMP2 and LIF were upregulated in the PD2 overexpressing Capan-1 cells (Supplementary Figure S3). These two proteins have been showed to be important for PDAC progression and stem cell maintenance. Earlier reports demonstrate that Bone Morphogenetic Protein 2 (BMP2) induces invasiveness in PDAC cells via Smad1 induction [22]. Furthermore, Leukemia inhibitory factor (LIF) is known to aid in the maintenance of stem cells [26].

## DISCUSSION

The current study seeks to investigate the expression pattern of a multifaceted molecule, PD2, in normal and PDAC tissues, and elucidate its functional importance in PDAC tumorigenesis and metastasis. Previous studies have been carried out to demonstrate that PD2 plays a role in cell cycle regulation and maintenance of mESCs, dictates histone methylation and is involved in ADM [12, 13, 15, 16]. However, no study has demonstrated its role in PDAC tumorigenesis and metastasis. The current study for the first time uncovers its role in tumorigenicity and metastasis.

The immunohistochemical analysis revealed that majority of the ducts present in the PDAC tissues had overexpression of PD2, whereas its expression was absent in the ducts of normal pancreatic tissues, thus emphasizing the importance of PD2 in PDAC. Normal tissues displayed a focal expression of PD2 in the acini suggesting that a basal level of PD2 is required to carry out normal function of acinar cells. In addition, it might also assist in the maintenance of the differentiated state of the acinar cell. Our previous and current studies suggest that the expressional variation of PD2 may be playing differential roles in different cell types.

Based on the differentiation status, the cell lines were classified into three groups such as well, moderately, or poorly-differentiated. The PDAC cell lines showed an increased expression of PD2 in the poorly-differentiated cell lines when compared to the moderately and well-differentiated cell lines. Similarly, the expression pattern of PD2 in the HPNE progression model suggests that PD2 is a potential oncogene whose expression is found to be increased in the transformed variants when compared to untransformed HPNE cells. This result indicated that PD2 overexpression may aid in cancer progression. In addition, it might aid in increasing the expression of oncogenes due to its role in transcription initiation and elongation. These results correspond to earlier observations of progressive increase in the expression of PD2 with increasing grades of PanIN lesions in Kras^G12D^; Pdx1-Cre (KC) and Kras^G12D^; Trp53^R172H/+^; Pdx1-Cre (KPC) mice [16]. The hPAF complex is crucial in playing a regulatory role in the transcription initiation and elongation of genes [23]. Therefore, it can be speculated that overexpression of PD2 leads to dysregulation in gene expression, resulting in cancer.

In our previous study, we have demonstrated that PD2 is overexpressed and is involved in the maintenance of pancreatic CSCs [9]. In the present study, we analyzed the tumorigenic effect of PD2 in both Capan-1 and SW1990 PDAC cells. Our results showed that overexpression of PD2 resulted in increased growth, colony formation as well as significant increase in tumor formation. This could be due to an increase in the expression of tumor promoting genes or decrease in tumor suppressing genes and/or an altered cell cycle. Indeed, overexpression of c-Myc and cyclin D1 was observed in PD2 overexpressed Capan-1 cells which support our earlier observation of PD2 regulating the expression of cyclins such as D1, E1, A1, A2 and B1, thus playing a key role in cell-cycle progression [14]. Additionally, the microarray data revealed that two important genes, LIF and BMP2, were overexpressed in PD2 overexpressing cells. These genes were essentially found to increase PDAC growth and invasiveness [22, 24]. These results suggest that PD2 is a positive regulator of oncogenesis in PDAC.

Previous studies have shown that poorly-differentiated pancreatic adenocarcinoma usually results in metastasis [25]. According to a recent study, it has been suggested that ∼70% of the PDAC patients die due to extensive metastasis [26]. We have investigated the expression of PD2 in metastatic sites (from the same patient) such as liver and diaphragm, and found that the expression of PD2 was increased in both sites. These results suggest that PD2 may play a potential role in the progression of PDAC and metastasis. Interestingly, PD2 overexpressed PDAC cells showed significantly increased migration which suggests that PD2 is also involved in the metastatic process. It is well known that PDAC cells metastasize to distant organ sites such as liver, peritoneal cavity, lung, lymph nodes, and intestinal lining. As expected, orthotopic implantation of PD2 overexpressing PDAC cells resulted in significantly higher metastasis to distant organs such as liver, stomach, diaphragm, peritoneal region and intestine.

The major cause of PDAC is the presence of activating point mutations in oncogenes such as Kras [27]. Therefore, an effort was made to perform a mutation analysis in the PDAC cell lines. The cell lines were found to contain no mutations in the coding sequence of the PD2 gene. This result could be similar to that of oncogenes including c-Myc, which are known to be devoid of mutations and whose aberrant expression leads to pathogenesis [28]. However, examination of PDAC patient samples is warranted for an in depth analysis of mutations of the PD2 gene. Sequence analysis of intronic, promoter and downstream regions of the gene may further give insight into any mutations present in the gene, which could affect its expression and function

It has been demonstrated that PAF complex requires the concerted action of all its components to exhibit its function in yeast. The PAF complex is reported to facilitate transcription elongation, mRNA processing and maturation [29]. However, a recent study demonstrated that the PAF1 depletion increased a group of nascent and mature transcripts in HCT116 cells through release of paused polymerase II from the promoter proximal site [30]. Under normal conditions, the human PAF complex is essential for transcription elongation through recruitment of FACT (facilitates chromatin transcription), a histone chaperone [31]. However, in our observation, PD2 plays a role independent of the PAF complex components in mESCs and pancreatic CSCs [9, 13]. Analysis of the effect of PD2 overexpression on the expression of other PAF complex members in PDAC cells showed a moderate upregulation of hCdc73; however, there was no difference in the expression of other PAF complex members. As Cdc73 or Parafibromin belongs to the PAF complex, it would be interesting to analyze the contribution of Cdc73 in tumor progression. Rather et al. showed that overexpression of Cdc73, a tumor suppressor resulted in downregulation of an oncogene such as c-Myc [32]. During cancer progression it is possible that overexpression of PD2 results in transcriptional elongation of certain genes via favorable chromatin structure and/or release of paused-RNA polymerase II from promoter proximal sites. Interestingly, our results showed that PD2 overexpression accelerates the expression of c-Myc in Capan-1 cells. c-Myc is an important oncogene whose overexpression results in increased cell proliferation, metastatic ability, downregulation of the expression of growth inhibitory genes and many others [33]. Interestingly, one of the previous reports showed that c-Myc expression accelerates the number of tumor-initiating-cells (TICs) in human keratinocytes transformed by Ras and IκBα [34]. Besides, c-Myc overexpression in residual cells could transform these dominant cells into TICs, thereby suggesting the importance of c-Myc in PDAC [35]. A recent study claims that c-Myc controls the self-renewal process in metastatic PDAC cells [36]. Interestingly, our studies reveal that PD2 overexpression leads to an increased SP/CSC population, thus corroborating with our previous reports [9]. This result suggests that overexpression of PD2 induces c-Myc expression which may be essential for the de-differentiation of cancer cells, thereby converting the cells into CSCs. At this point the possible mechanism could be that PD2 overexpression leads to increased LIF which may leads to increased c-Myc, thus resulting in oncogenesis. However, further analysis is required to understand the complete mechanism.

Collectively, our studies showed the overexpression of PD2 in the malignant ducts compared to undetectable levels in the ducts of the normal pancreas, suggesting the significance of PD2 in PDAC progression. Also, expression of PD2 is observed in the primary tumors and matched metastatic sites such as liver and diaphragm, denoting that PD2 may be an essential player during metastasis (Figure 6D). For the first time, we have demonstrated that increased expression of PD2 accelerates tumorigenesis and metastasis in PDAC cells. Our work has further shown that PD2 controls the expression of oncogenes such as c-Myc and cyclin D1, which are involved in promoting PDAC. In a nutshell, our results suggest that PD2 is an essential member of the oncogene family, and its association with PDAC and metastasis necessitates further studies on the structural and functional characterizations of PD2 which may facilitate the design of various therapeutic strategies.

## MATERIALS AND METHODS

### Ethics statement

Tissue samples were obtained through the Rapid Autopsy Program (RAP) at UNMC. An informed consent was signed prior to tissue collection from all the patients. The Whipple samples were obtained from the UNMC tissue bank after the Institutional Regulatory Board approval number IRB#186-14.

### Tissue specimen and cell lines

A total of 43 formalin fixed and paraffin embedded (FFPE) PDAC tissues were obtained from patients who were eligible for the Whipple procedure. In addition, 3 tissue arrays were also obtained from the RAP which contained tissues from the normal pancreas (*n* = 9 spots) and also the primary and metastatic sites of the PDAC patients (*n* = 80 spots in total). The details of RAP at UNMC are well described in our previous publication [37]. The PDAC tissue array containing primary tumor spots were classified as well-differentiated adenocarcinoma (*n* = 2), moderately-differentiated carcinoma (*n* = 27) and poorly-differentiated carcinoma (*n* = 16).

All the PDAC cell lines used in the present study such as Panc-1, AICPC-1, AsPC-1, BxPC3, Capan-1, Capan-2, CD11, CD18/HPAF, FG, HPAC, Hs766T, MiaPaCa-2, Panc-89, QgP1, S2CP9, Suit2 and T3M4 were originally obtained from American Type Culture Collection (ATCC). These PDAC cell lines were regularly cultured in respective culture medium (RPMI or DMEM) supplemented with 10% fetal calf serum and antibiotics (penicillin and streptomycin 100U/ml) in the presence of 5% CO_2_ at 37°C.

This study also used hTERT-HPNE (Human Pancreatic Nestin-Expressing) cells, a line of telomerase-immortalized normal human pancreatic ductal cells [18]. hTERT-HPNE cells can be transformed by the stepwise introduction of oncogenes designed to mimic PDAC progression, including oncogenic Kras (carrying G12D mutation), HPV16 E6 and E7 proteins (to abrogate p53 and RB), and SV40 small t antigen (to inhibit PP2A). These cell lines were grown in medium D which contains three volumes of DMEM and one volume of medium M3, with the mixture supplemented with 5% fetal calf serum and 10 ng/mL EGF.

### Lentiviral-mediated infection

Approximately 1.5 × 10^6^ HEK cells were seeded in a 10 cm plate containing 5% RPMI media and 10 ml L-glutamine. The following day 6 ml of serum-free DMEM media was added to the cells. Lenti-viral transfection was carried out 3-4 hours after the media change. The pPACKH1 Lentivector packaging kit (System biosciences, CA) plus lentiviral vector carrying PD2 was used to perform the transfection. For this, 20 μl of pPACKH1 and 30 μl of lipofectamine were added to 2 μg of the empty vector and the PD2 overexpression vector. To this, 20 μl plus reagent was added, gently tapped and incubated. Slowly the mixture was added drop by drop into the plates. Meanwhile, 100,000 parental PC cells were seeded in a 6 well plate. 48 hours after transfection in HEK cells the viral supernatant was collected and added to the parental PC cells along with polybrene (10 mg/ml). Approximately, 10 μl of polybrene was added to 1 ml of viral supernatant. After 48–72 hours of transduction, the GFP expression was detected in the cells.

### Immunohistochemistry

Immunohistochemistry (IHC) analysis was performed as described previously [38]. For IHC, our in-house generated anti-PD2 mouse monoclonal antibody was used [14]. PD2 expression in human tissues was scored by pathologists from UNMC on a double blind condition. The intensity of PD2 expression was graded on a scale of 0–3 (0− no staining, 1+ weakly positive, 2+ moderately positive and 3+ strongly positive). The percentage of PD2-positive staining in PDAC ducts was scored in a range of 1–4 (1: 0–25% positive cells; 2: 26–50%; 3: 51–75% positive; and 4: 76–100% positive cells.). A composite score (CS) was calculated by multiplying the intensity and positivity which ranges between 0 and 12. The tissues were categorized as negative (CS = 0) and positive (CS ≥ 1) based on their composite scores.

### RNA extraction and PCR

Total RNA was extracted and PCR was performed using cDNA as described previously [9]. Primers used in this study are (i) PD2 RT-PCR primer; Forward1-(5′-TTCCTCGGATCAGGCGTCCC-3′) Reverse1-(5′-CTGGGACTCAGTCACTGTCACTA-3′), (ii) PD2 qRT-PCR primer; Forward2-(5′-TGATTCAGACAGCGGCA-GCAATGG-3′), Reverse2-(5′-TTGCT-GCCGCTGTCTGAATCATTG-3′).

### Western blot analysis

Western blot (WB) analysis was carried out using standard procedures as described previously [9]. The membrane was probed overnight with antibodies such as rabbit polyclonal antibodies anti-hPaf1, anti-Parafibromin, anti-Ctr9, (Bethyl Laboratories, TX) and anti-c-Myc (Sigma). β-actin was used as a loading control. To quantify the band intensity, densitometry was performed using the ImageJ program (http://rsb.info.nih.gov/). For relative protein level, the band intensity was first normalized to the relative β-actin protein level, then to their respective control groups. The control ratio was set to equal 1.

### Cell proliferation assay

In order to compare the effect of PD2 on growth rate, cell proliferation was determined in PD2 overexpressed and respective control cells using WST-1 assay [39].

### Migration and clonogenic assay

To perform the migration assay, 0.1 × 10^6^ and 1 × 10^6^ of SW1990 and Capan-1 PDAC cells were used and the assay was carried out as described previously [40]. For clonogenic survival assay, approximately, 500 control and PD2 overexpressing PDAC cells per 10 cm plates were seeded in triplicates in 10 ml of 10% serum containing media. Cells were fed with fresh media every 3 days. After two weeks, the media was aspirated from the plates, cells were washed twice with PBS and fixed using ice cold methanol. After fixation the cells were stained using 0.2% crystal violet and destained with excess water. The total number of colonies was counted, averaged and the representative pictures were presented.

### Orthotopic implantation

Four to six weeks old nude mice (*n* = 6 per group) were purchased from Harlan Sprague dawley, Indianapolis, IN and were housed at UNMC animal facility in pathogen free conditions and fed with sterile water and ad libitum. The mice were treated in accordance with the IACUC guidelines. The cells were washed using PBS, counted using the haemocytometer and were resuspended at a concentration of 0.5 × 10^6^ cells/μl. The mice were anesthetized using ketamine and xylazine mixture (4:1). A small incision was made in the left abdominal flank and the cell suspension was slowly injected into the head of the pancreas. The animals were monitored periodically. After six weeks, animals were sacrificed and tumor weights were measured. All the major organs and potential sites were excised and analyzed for metastasis. For micro and macrometastasis, the resected organs were sectioned and processed for histochemical analysis.

### Microarray analysis

RNA was extracted from both control and PD2 overexpressing cells. The quality of RNA was determined using a bioanalyzer and the RNA integrity number (RIN = 9.9) was measured. Approximately 1 μg RNA was used for the microarray analysis. Microarray was performed using Affymetrix GeneChip Human Gene 2.0ST array. The differential expression analysis was performed using the online software-ingenuity pathway analysis (Qiagen).

### CSC/SP analysis

To determine the shift in the percentage of CSC population, both the control and PD2 overexpressed cells were subjected to FACS analysis. The percentage of SP/CSC population was analyzed as described previously [9].

### Mutation analysis

The PD2 coding region was amplified in 2 overlapping fragments from 13 different PDAC cell lines (Panc-1, MiaPaCa-2, BxPC-3, Capan-1, HPAF, SW1990, T3M4, Capan-2, CD18, CFPAC-, Panc-89, and Colo357). The amplified regions were then sequenced and determined by dye-labeled terminator chemistry using the ABI 3700 automated DNA sequencer (Applied Biosystems, Foster City, CA) at Macrogen USA, Rockville, MD. Data generated i.e., the sequences were analyzed using the Mutation Surveyor^™^ version 3.01 (SoftGenetics, LLC).

### Statistical analysis

For the RAP tissue scoring, Wilcoxon rank sum test was used to compare composite scores (CS) between groups. For paired samples, Wilcoxon signed rank test was employed. We also categorized CS score as 0, 1–4, 5–8, and 9–12 and compared by tissue type and differentiation status with chi-square tests (Fisher's exact test was used for small sample situations). Paired samples were compared with a test of symmetry (similar to McNemar's test). For Whipple samples, Fisher's test was used to determine an association between the average composite score and cancer vs. normal. *P*-values less than 0.05 were considered to be statistically significant.

## SUPPLEMENTARY MATERIAL FIGURES


