# Supplementary material for: Overexpression of PD2 leads to increased tumorigenicity and metastasis in pancreatic ductal adenocarcinoma
Source: Oncotarget. 2015 Dec 12;7(3):3317–31. doi: 10.18632/oncotarget.6580 (PMC4823108; doi:10.18632/oncotarget.6580)
Supplement: Supplementary file 1 [file oncotarget-07-3317-s001.pdf]

Overexpression of PD2 leads to increased tumorigenicity and metastasis in pancreatic ductal adenocarcinoma

Supplementary Materials

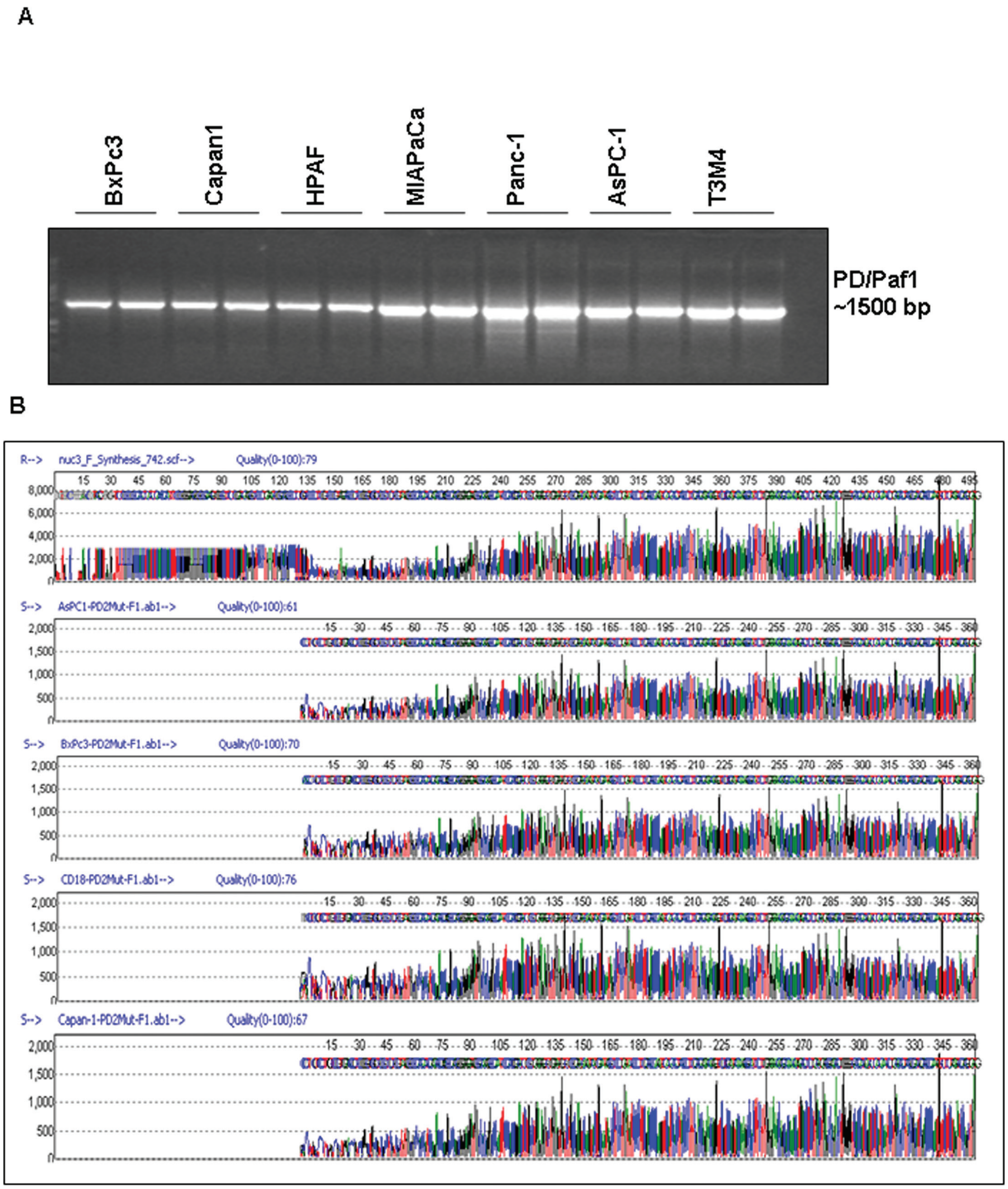

Supplementary Figure S1: Analysis of the mutation status of PD2/hPaf1 in pancreatic cancer cell lines. (**A**) Representation of amplified PD2 cDNA from pancreatic cancer cell lines. (**B**) Representative results showing direct sequencing of a part of the PD2/hPaf1 sequence in a few pancreatic cancer cell lines, ASPC-1, BxPc3, CD18/HPAF and Capan-1. Analysis did not reveal any mutations in the cell lines.

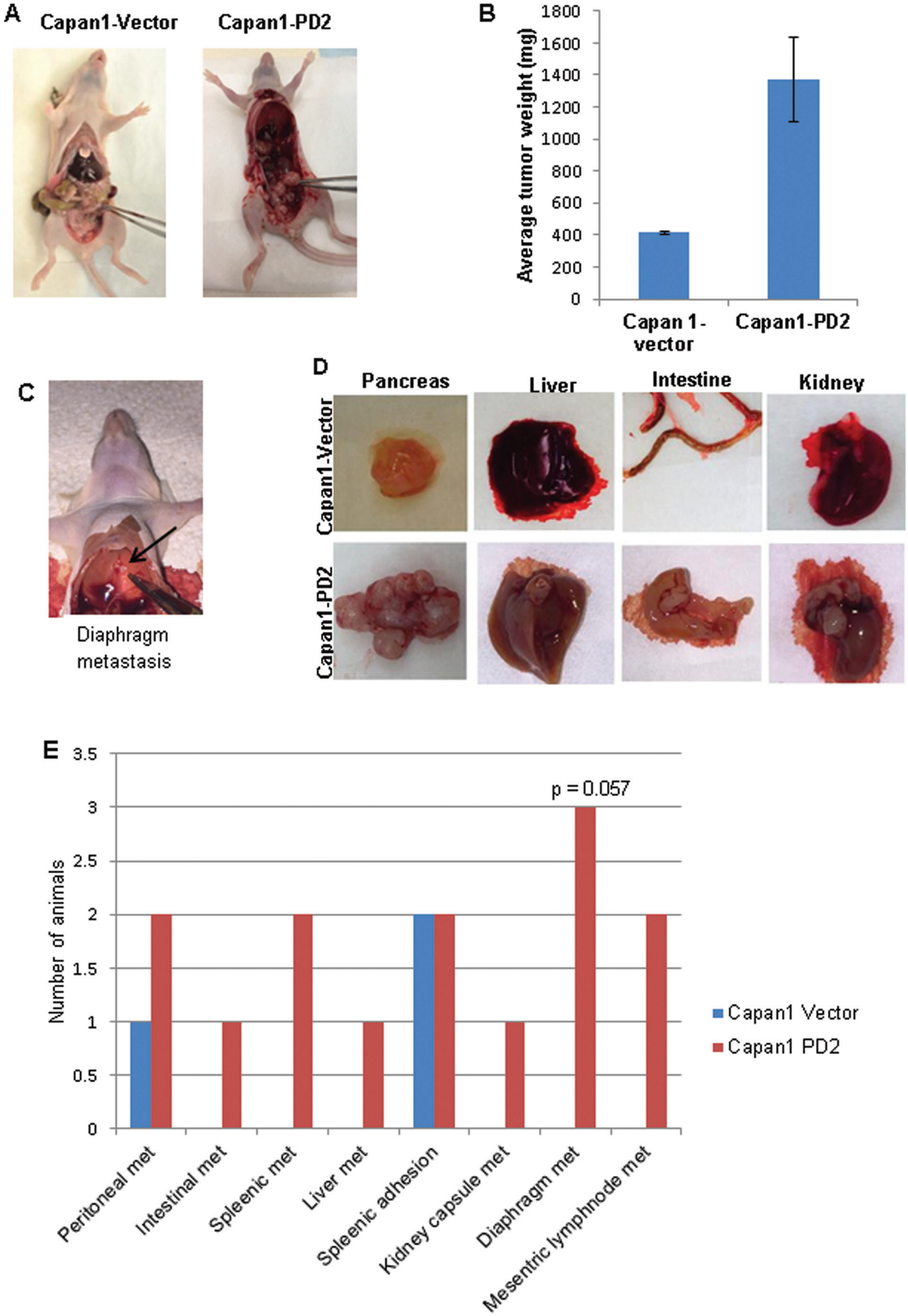

Supplementary Figure S2: PD2 overexpression promotes increased tumor formation and metastasis in nude mice. (**A**) Overexpression of PD2 in Capan-1 cells led to increased tumor formation in the pancreas. The nude mouse was dissected and represented to show the presence of large tumor formed at the primary site. (**B**) The weights of the tumors formed were compared and are presented in the bar diagram. (**C**) A representative image of animal (injected with Capan-1-PD2 cells) having diaphragm metastasis is shown. (**D**) Representation image of primary and metastatic sites (Liver, Intestine and Kidney). (**E**) comparison of the metastatic nature of Capan 1 vector and PD2 overexpressed cells.

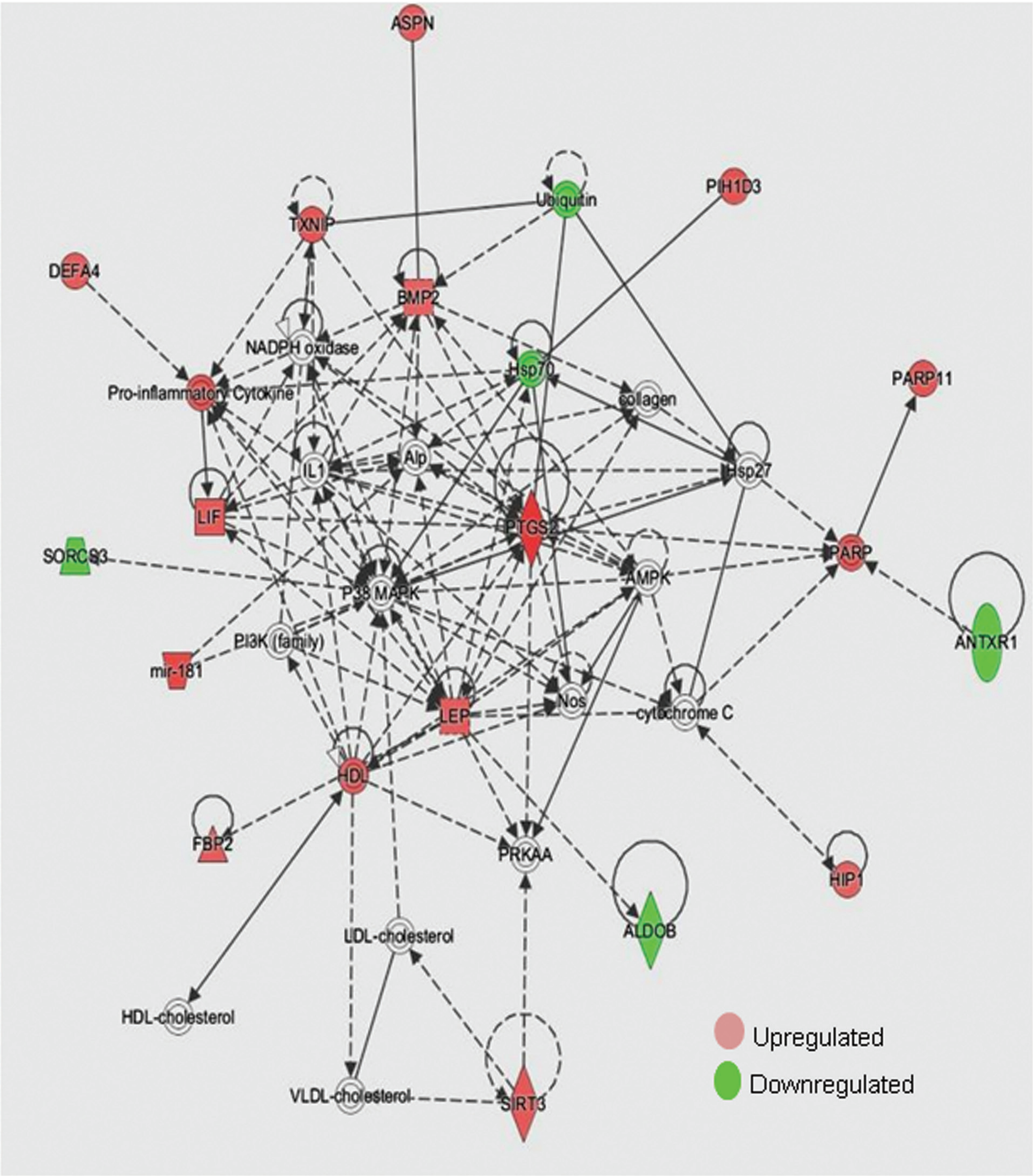

Supplementary Figure S3: Microarray analysis performed using Capan-1-control and PD2 overexpressing cells revealed that PD2 overexpression led to upregulation of LIF and BMP-2.
